# Spontaneous Tumor Lysis Syndrome Due to Endometrial Carcinoma

**DOI:** 10.7759/cureus.7220

**Published:** 2020-03-09

**Authors:** Juan Jose Chango Azanza, Vinay Mathew Thomas, Paola Michelle Calle Sarmiento, Meghana Singh, Swetha Ann Alexander

**Affiliations:** 1 Internal Medicine, University of Connecticut, Farmington, USA; 2 Internal Medicine, University of Connecticut , Farmington, USA; 3 Internal Medicine, Catholic University of Cuenca, Cuenca, ECU; 4 Internal Medicine, University of Connecticut, Hartford, USA

**Keywords:** spontaneous tumor lysis syndrome, endometrial cancer, acute kidney injury, tumor lysis in solid tumors

## Abstract

Spontaneous tumor lysis syndrome (TLS) is a rare condition in solid tumors, particularly in endometrial carcinoma. Spontaneous TLS occurs without the use of cytotoxic therapy but is observed particularly in hematologic malignancies. Given the high morbidity and mortality associated with spontaneous TLS, it is crucial to identify and treat it promptly. There have been only four cases of spontaneous TLS reported to date in the literature from a uterine source. We present a 59-year-old female with a recently diagnosed endometrial carcinoma with neuroendocrine features by dilation and curettage who presented to the hospital with somnolence, decreased oral intake, and lower abdominal pain of three days duration. She was found to have sepsis secondary to endometritis and spontaneous tumor lysis syndrome by clinical and laboratory definitions (hyperkalemia, hyperphosphatemia, hyperuricemia, and hypocalcemia). Signs of disease progression were found such as worsening retroperitoneal lymphadenopathy that corresponded with the suspected increased tumoral activity. We report the case of a solid tumor (endometrial) presenting with spontaneous TLS, which highlights the importance of the early identification and initiation of treatment.

## Introduction

Tumor lysis syndrome is a life-threatening condition where the extensive destruction of tumor cells leads to the release of potassium, uric acid, and phosphate into the blood, leading to life-threatening complications such as renal failure, metabolic derangements, seizures, and death [1]. TLS occurs mostly after the initiation of cytotoxic therapy. Spontaneous TLS has been reported in the past and has been seen mostly in hematologic malignancies. However, spontaneous TLS in solid tumors is a rare occurrence. Furthermore, there are case reports of spontaneous tumor lysis reported in gynecologic malignancies [1]. Therefore, it is crucial to suspect spontaneous tumor lysis syndrome in patients with a history of malignancy who present with hyperkalemia, hyperuricemia, hyperphosphatemia, hypocalcemia, kidney dysfunction, muscle cramps, tetany, nausea, vomiting, change in mental status, cardiac arrhythmias, syncope, or sudden death. Given the high morbidity and mortality with TLS, identifying and treating it promptly is beneficial. We present a case of spontaneous tumor lysis syndrome due to endometrial carcinoma.

## Case presentation

We present the case of a 59-year-old female with a past medical history of essential hypertension, type II diabetes mellitus, obesity, and a recently diagnosed, poorly differentiated adenocarcinoma of the endometrium with neuroendocrine features. She presented to the emergency department with a history of somnolence, decreased oral intake, decreased energy, and lower abdominal pain of three days duration. Two weeks before presenting to the hospital, the patient was evaluated as an outpatient for post-menopausal bleeding and lower abdominal pain. A computed tomography (CT) scan of the abdomen and pelvis showed a distended uterus, an irregular hypodensity, 3.5 cm in diameter; an increased endometrial thickness of 4.3 cm, and retroperitoneal lymphadenopathy. She underwent a dilation and curettage, which yielded abundant blood and fragments of malignant, poorly differentiated carcinoma with neuroendocrine features and necrosis.

**Figure 1:**
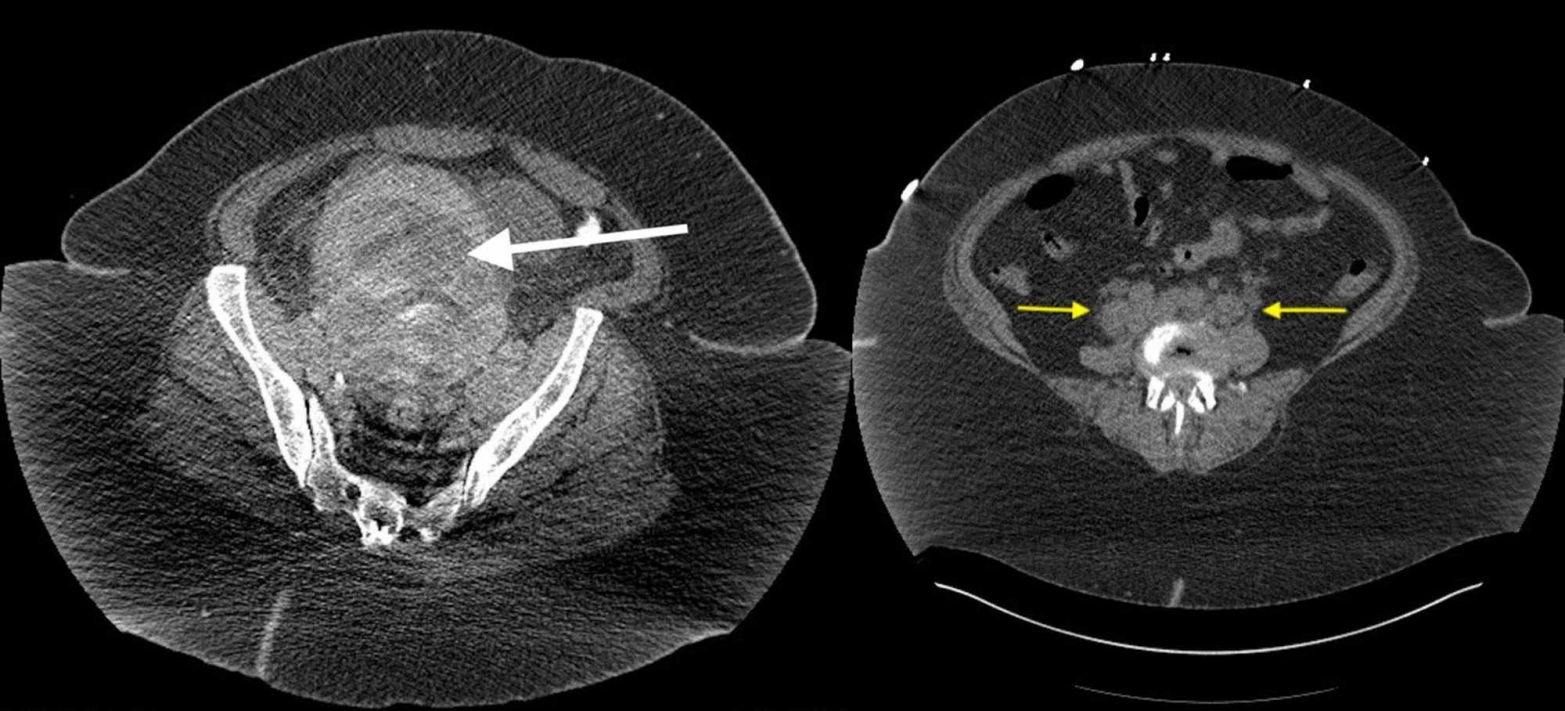
Computed tomography of the abdomen and pelvis showing a large uterine (white arrow) and retroperitoneal lymphadenopathy (yellow arrows)

On initial evaluation in the hospital, she was hypotensive and tachycardic. Her physical exam showed signs of hypovolemia (dry mucous membranes, flat jugular veins, dry skin, hypotension, and tachycardia) and tenderness to palpation in the lower abdomen. Her laboratory workup was relevant for leukocytosis (32.000/uL) with neutrophilia, normocytic anemia (10.9 g/dL), thrombocytosis (476 K/uL), acute kidney injury (creatinine 5.8 mg/dL with creatinine at baseline close to 0.9 mg/dL), hyperkalemia (5.8 mmol/L), hyperphosphatemia (7.1 mg/dL), hypocalcemia (6.1 mg/dL), elevated lactate dehydrogenase (2471 U/L), hyperuricemia (22.7 mg/dL), lactic acid elevation (5.1 mmol/L), and an elevated procalcitonin (4.86 ng/mL). A CT scan of the abdomen showed gas within the uterus and worsening retroperitoneal lymphadenopathy when compared to the previous CT scan done a few weeks earlier (Figure 1).

The patient received intravenous fluid resuscitation, resulting in an improvement in hypotension. Given the recent uterine instrumentation, treatment for sepsis secondary to endometritis was started. She was deemed not suitable for surgery given her acute deterioration and was recommended medical management. The patient met laboratory and clinical criteria for tumor lysis syndrome by the Cairo-Bishop definition. Given her severe renal dysfunction, nephrology was consulted and recommended rasburicase for the treatment of tumor lysis syndrome, which improved her uric acid levels. Unfortunately, she became oliguric and had worsening kidney function, requiring renal replacement therapy. Her hospital course was complicated by respiratory failure requiring intubation, worsening hypotension requiring pressor support, and multiorgan failure, among other complications. Finally, the patient and her family decided to transition to comfort care and the patient expired four days after admission.

## Discussion

Tumor lysis syndrome is a life-threatening medical condition in which tumor cells are destroyed or replicated rapidly, causing a release of intracellular contents into the bloodstream. Spontaneous tumor lysis syndrome in solid tumors is rare. The incidence of TLS has been reported as 42% although the incidence of clinically significant TLS is approximately 6% [2]. TLS after cytotoxic therapy has been reported in nonhematologic solid tumors such as breast cancer, lung cancer, small cell germinoma, neuroblastoma, ovarian cancer, urothelial cancer, metastatic colorectal cancer, and others [1]. However, spontaneous TLS is a very infrequent finding, especially in patients with gynecologic malignancies in which treatment has not been used. The pathogenesis of TLS explains most of the laboratory abnormalities found in these patients. The degradation of purines raises the levels of uric acid released into the bloodstream. The increased amount of uric acid in the kidneys causes precipitation in the renal tubules, vasoconstriction, decreased renal blood flow, and inflammation that result in acute kidney injury. Cell lysis causes the spilling of intracellular contents, such as potassium, phosphate, and uric acid, which cause hyperkalemia, hyperphosphatemia, and hyperuricemia. 

A high proliferative rate, large tumor burden, high sensitivity to cytotoxic chemotherapy, radiation therapy, cytolytic antibody therapy, use of corticosteroids, dehydration or inadequate hydration during treatment, oliguria, or nephropathy increases the risk of developing TLS [3]. Risk stratification is possible by the analysis of risk factors by clinical and laboratory findings, such as the tumor lysis risk and mass bulk into low, moderate, and high risk [3]. Patients with hematologic malignancies have high indexes of replication and thus develop TLS more often. Certain chemotherapy agents, such as venetoclax, obinutuzumab, and others, cause more prominent cell destruction and are associated with increased rates of tumor lysis syndrome occurrence [3].

The clinical manifestations in spontaneous TLS include, but are not limited to, cardiac arrhythmias, renal failure, seizures, dehydration from vomiting or diarrhea, encephalopathy, muscle cramps, tetany, syncope, and death [1]. The diagnosis of tumor lysis syndrome can be made by using the Cairo-Bishop definition of TLS, which is widely recognized as the main tool for diagnosis (Table 1). Clinical TLS is defined as laboratory TLS plus one or more of the following that was not directly or probably attributable to a therapeutic agent: increased serum creatinine concentration (≥1.5 times the upper limit of normal (ULN)), cardiac arrhythmia/sudden death, or a seizure [2]. The management of TLS consists mostly of intravenous hydration, electrolyte disorder correction and management, the use of rasburicase, renal replacement therapy, and other interventions.

**Table 1:** Cairo-Bishop definition of laboratory tumor lysis syndrome

Cairo-Bishop Definition			
Element	Value	Change from baseline	Our patient
Potassium	≥6 mEq/L (or 6.0 mmol/L)	25% increase	5.8 mmol/L
Uric acid	≥8 mg/dL (476 micromol/L)	25% increase	22.7 mg/dL
Phosphorus	≥4.5 mg/dL (1.45 mmol/L)	25% increase	7.1 mg/dL
Calcium	≤7 mg/dL (1.75 mmol/L)	25% decrease	6.1 mg/dL
Laboratory tumor lysis syndrome defined by any 2 of 4 findings			

In our review of the literature, we found four reported cases of spontaneous TLS related to endometrial cancer. Harada et al., in 2015, reported the case of a 59-year-old female with multiple masses in the uterus and pathology positive for a dedifferentiated endometrial adenocarcinoma who met criteria for TLS without receiving cytotoxic therapy [4]. Two cases of spontaneous TLS were reported by Berger et al. in 2017 [5]. Case 1 describes a 33-year-old female with a mass in the uterine fundus and bilateral ovarian masses positive for metastatic endometrioid endometrial adenocarcinoma who met criteria for TLS without treatment of the disease. Case 2 described a 65-year-old female with a history of diffuse large B cell lymphoma (DLBCL) treated with chemotherapy who was found a primary uterine malignancy with cervical extension. Unfortunately, given the history of DLBCL on current cytotoxic therapy, it is difficult to say whether this case was spontaneous TLS secondary to a uterine malignancy only. Vivek et al. reported the case of a 58-year-old female with a large pelvic mass with peritoneal carcinomatosis, pulmonary nodules, pleural effusions, and mediastinal lymphadenopathy positive for metastatic uterine leiomyosarcoma [6]. Ahmed et al. in 2019 reported the case of a 79-year-old female with spontaneous TLS due to a metastatic undifferentiated endometrial stromal sarcoma [7].

Tumor lysis syndrome has a high mortality rate. A study showed that the mortality of TLS in hematologic malignancies is 19% and 32.3% in solid tumors (p<0.001) [8]. The authors of this study concluded that there was a higher rate of patients requiring mechanical ventilation and increased mortality in patients with TLS from solid tumors when comparing to hematologic malignancies. Therefore, given the high morbidity and mortality seen in TLS cases, it is crucial to have a high index of clinical suspicion to detect this condition and treat it promptly.

## Conclusions

Tumor lysis syndrome rarely occurs spontaneously in solid tumors. This case reflects the importance of considering tumor lysis syndrome as a cause of kidney dysfunction, seizures, or metabolic derangements, such as hyperkalemia, hyperphosphatemia, hyperuricemia, and hypocalcemia in a patient with a solid tumor. Evidence of disease progression is a clue for spontaneous TLS when these metabolic derangements are found. Given the high morbidity and mortality with TLS, it is crucial to identify and treat it promptly.
